# Vascular Access Perspectives in Patients After Kidney Transplantation

**DOI:** 10.3389/fsurg.2021.640986

**Published:** 2021-04-28

**Authors:** Krzysztof Letachowicz, Mirosław Banasik, Anna Królicka, Oktawia Mazanowska, Tomasz Gołębiowski, Hanna Augustyniak-Bartosik, Sławomir Zmonarski, Dorota Kamińska, Magdalena Kuriata-Kordek, Magdalena Krajewska

**Affiliations:** ^1^Department of Nephrology and Transplantation Medicine, Wroclaw Medical University, Wrocław, Poland; ^2^Faculty of Medicine, Wroclaw Medical University, Wrocław, Poland

**Keywords:** vascular access, arteriovenous fistula, end-stage kidney disease, hemodialysis, hemodialysis catheter, kidney transplantation, vascular access after transplantation

## Abstract

**Introduction:** More attention has been paid to the influence of arteriovenous fistula (AVF) on the cardiovascular system. In renal transplant recipients, some beneficial effect of an elective vascular access (VA) ligation was observed in patients with a high AVF flow. However, this strategy is not widely accepted and is in contradiction to the rule of vasculature preservation for possible future access. The aim of our study is to elucidate the vascular access function and VA perspective in the kidney transplantation (KTx) population.

**Materials and Methods:** KTx patients with a stable graft function were recruited to participate in this single center observational study (NCT04478968). The measurement of VA flow and vessel mapping for future vascular access was performed by a color Doppler ultrasound. The study group included 99 (63%) males and 58 (37%) females; the median age was 57 (IQR 48–64) years. The median time from the transplantation to the baseline visit was 94 (IQR 61–149) months. Median serum creatinine concentration was 1.36 (IQR 1.13–1.67) mg/dl.

**Results:** Functioning VA was found in 83 out of 157 (52.9%) patients. The sites were as follows: snuffbox in six (7.2%), wrist in 41 (49.4%), distal forearm in 18 (21.7%), middle or proximal forearm in eight (9.6%), upper-arm AV graft in one (1.2%), and upper-arm AVFs in nine (10.8%) patients, respectively. Blood flow ranged from 248 to 7,830 ml/min; the median was 1,134 ml/min. From the transplantation to the study visit, 66 (44.6%) patients experienced access loss. Spontaneous thrombosis was the most common, and it occurred in 60 (90.9%) patients. The surgical closure of VA was performed only in six (4%) patients of the study group with a functioning VA at the time of transplantation. Access loss occurred within the 1st year after KTx in 33 (50%) patients. Majority (50 out of 83, 60.2%) of the patients with an active VA had options to create a snuffbox or wrist AVF on the contralateral extremity. In a group of 74 patients without a functioning VA, the creation of a snuffbox or wrist AVF on the non-dominant and dominant extremity was possible in seven (9.2%) and 40 (52.6%) patients, respectively. In 10 (13.1%) patients, the possibilities were limited only to the upper-arm or proximal forearm VA on both sides. Access ligation was considered by 15 out of 83 (18.1%) patients with a patent VA.

**Conclusions:** In the majority of the patients, vascular access blood flow was below the threshold of the negative cardiovascular effect of vascular access. Creation of a distal AVF is a protective measure to avoid a high flow and preserve the vessels for future access. The approach to VA should be individualized and adjusted to the patient's profile.

## Introduction

Arteriovenous fistula (AVF) is considered as the optimal vascular access (VA) for hemodialysis, but its broad use is limited by multiple factors. The most important ones are comorbidities, quality of the vascular bed, and organizational issues (1, 2). The latter is illustrated by huge differences in the proportion of patients starting dialysis with use of a catheter and the percentage of forearm AVFs (3, 4). However, for a large group of patients, the dialysis is not an ultimate option and they undergo kidney transplantation (KTx). In this group, dialysis access is needed temporarily. In patients who have a transplanted kidney, the type of VA has not influenced the mortality and all-cause allograft loss (5). In recent years, the interest in VA in KTx recipients is growing. The main research topics are: cardiac impact, potential renal function preservation, transplantation-dialysis transition, patients and professionals' opinion (6–9). The problems of vascular access for dialysis could not be perceived until the very late phases of renal failure (despite regular follow up) in patients with a failing renal transplant resulting in a more frequent use of a catheter (10). Worse VA quality metrics were observed in other cohorts (11). The next important issue seems to be the AVF impact on the cardiovascular system. Beneficial effect in the form of a left ventricular mass reduction was observed after an elective AVF ligation in patients with a high vascular access flow (Qa). However, this strategy is not widely accepted. The question “to ligate or not to ligate” is still unanswered (12), but the aim of vessel preservation is essential (13). Vessel protection is one of the cornerstones of the End-Stage Kidney Disease plan (14). The aim of our study is to elucidate the AVF function and vascular access perspective in the KTx population.

## Materials and Methods

Between July and October 2020, 157 patients at least 12 months after KTx were recruited to participate in a prospective observational study. The aim of the study was to recruit 75 patients with a functional AVF and 75 without VA. The study protocol was approved by the Institutional Ethical Committee at Wroclaw Medical University (KB-43/2020) and the study was registered (NCT04478968). All patients were served by the Department of Nephrology and Transplantation Medicine in Wroclaw Medical University. Written informed consent was obtained from all patients. Apart from the routine visit, all patients were assessed by an interventional nephrologist with an experience in access creation and monitoring. Clinical data were collected. They included information on the demographics, comorbidities, vascular access history and function, and routine laboratory data. The presence of a heart disease was defined as a history of coronary artery disease, valvular heart disease, or arrhythmia. Patients with dyspnea, peripheral edemas, or crackles on auscultation were classified as symptomatic. Patients were asked for their opinion on the ligation of VA. Clinical assessment of vascular access was performed. The AVFs were inspected, palpated, and the character of bruit was assessed. Finally, an arm elevation test was done (15). On the basis of physical examination, the functioning AVFs were categorized into four groups, from dysfunction to suspicion of high-flow. The vasculature of both upper extremities was assessed with an ultrasound. All ultrasound examinations were performed with the Samsung HS50 system. The extremities without a functioning arteriovenous fistula were screened to find the most distal possible site for future vascular access. Minimal vessel diameter suitable for AVF creation was set on 1.5 mm (and 2 mm in case of atherosclerosis) for an artery and 2 mm for a vein. Arteriovenous fistula flow was assessed in accordance with the guidelines (16). The characteristic of the studied cohort was summarized in Table 1. Most of the patients had been treated with hemodialysis before KTx. Majority of patients (151, 96.2%) received a graft from a deceased donor.

**Table 1:** Characteristics of the study group.

**Characteristic**	**All patients (*n* = 157)**
Age, median (IQR) [years]	57 (48–64)
Male, n (%)	99 (63%)
BMI, median (IQR) [kg/m^2^]	25.95 (23.83–29.29)
**Primary kidney disease, n (%)**	
Glomerulonephritis	93 (59.2%)
ADPKD	25 (15.9%)
DM	8 (5.1%)
Reflux nephropathy	7 (4.4%)
Other	21 (13.4%)
Unknown	3 (1.9%)
Duration between study visit and transplantation, median (IQR) [months]	94 (61–149)
Duration between study visit and RRT initiation, median (IQR) [months]	143 (97–207)
First transplantation, n (%)	133 (84.7%)
Serum creatinine concentration, median (IQR) [mg/dl]	1.36 (1.13–1.67)
Heart disease, n (%)	53 (33.7%)
Diabetes mellitus, n (%)	35 (22.3%)
Charlson comorbidity index, median (IQR)	4 (3–5)
Smoking, current or previous, n (%)	80 (50.9%)
Patency of AVF, n (%)	83 (52.8%)
History of dialysis catheter insertion, n (%)	69 (43.9%)
**Medications, n (%)**	
Steroids	152 (96.8%)
Tacrolimus	129 (82.2%)
Cyclosporine	28 (17.8%)
Mycophenolate	128 (81.5%)
mTOR inhibitors	7 (4.4%)
Azathioprine	6 (3.8%)
Antihypertensive	142 (90.4%)
Statins	60 (38.2%)
Antiplatelet/anticoagulants	39 (24.8%)

*BMI-body mass index; ADPKD-adult dominant polycystic kidney disease; RRT-renal replacement therapy; AVF-arteriovenous fistula*.

### Statistical Analysis

All statistical analyses were performed using the Statistical 13.3 (StatSoft, Poland). The normality of data distribution was tested with the Shapiro–Wilk test. Categorical variables were presented as proportions and compared using the Chi Square test. Continuous data were presented as median and interquartile range. Non-parametric Mann–Whitney *U* and Kruskall-Wallis tests were used for numerical data analysis. A *p*-value below 0.05 was considered statistically significant.

## Results

At the time of transplantation, 142 (90.4%) patients had been dialyzed with AVFs, three (1.9%) with AVG, three (1.9%) with a tunneled catheter, seven (4.4%) patients were on a peritoneal dialysis, and two (1.3%) patients had been transplanted preemptively. Two females had had a functioning vascular access on both upper extremities (dysfunctional AVFs and forearm AVF or AVG on the opposite site, respectively). Only four (2.5%) patients had never experienced any attempt of AVF creation: one patient underwent preemptive transplantation from a living donor and three patients were on a peritoneal dialysis. Functioning arteriovenous VA was present in 148 (94.2%) patients. Vascular access was located in the distal part of the extremity, in the proximal forearm, and on the upper arm in 120 (81.1%), eight (5.4%), and 20 (13.5%) patients with a proportion of first access of 72.5, 12.5, and 35%, respectively.

From the transplantation to the study visit, 66 (44.6%) patients experienced access loss. Spontaneous thrombosis was the most common, and it occurred in 60 (90.9%) patients. In three individuals, surgical revision was necessary. The surgical closure of VA was performed only in six patients (4% of the study group with a functioning AVF at the time of KTx). Access loss occurred within the 1st year after KTx in 33 (50%) patients. In 16 (24.2%) patients, access thrombosis was diagnosed in the 1st month after KTx. The comparison of groups with a functioning VA and after VA with spontaneous thrombosis is presented in Table 2.

**Table 2:** Comparison of patients with a functioning and thrombosed AVF.

**Characteristic**	**Patent (82)**	**Thrombosis (60)**	***P*-value**
Age, median (IQR) [years]	59 (48–64)	54.5 (45–63)	0.22
Male, n (%)	57 (69.5%)	36 (60%)	0.24
BMI, median (IQR) [kg/m^2^]	26.19 (24.19–29.41)	25.63 (23.6–29)	0.31
Serum creatinine concentration, median (IQR) [mg/dl]	1.4 (1.13–1.64)	1.36 (1.14–1.71)	0.95
Duration between study visit and transplantation, median (IQR) [months]	76 (48–115)	122.5 (73–189)	<0.0001
Duration between study visit and RRT initiation, median (IQR) [months]	126.5 (84–178)	168.5 (112–229)	0.0047
First transplantation, n (%)	68 (82.9%)	55 (91.7%)	0.13
Prior diabetes mellitus, n (%)	20 (24.4%)	13 (21.7%)	0.70
Prior heart disease, n (%)	32 (39%)	16 (26.7%)	0.12
Charlson comorbidity index	4 (3–6)	4 (3–5)	0.75
Smoking, current or previous, n (%)	42 (51.2%)	33 (55%)	0.65
Office SBP, median (IQR) [mmHg]	154 (142–169)	154 (134–168)	0.51
Office DBP, median (IQR) [mmHg]	92 (83.5–99.5)	98 (92–105)	0.0082
Number of antihypertensive drugs 0 1 2 3 >3	7 (8.5%) 18 (21.9%) 29 (35.4%) 22 (26.8%) 6 (7.3%)	7 (11.7%) 15 (25%) 24 (40%) 9 (15%) 5 (3.3%)	0.56
History of dialysis catheter insertion, n (%)	34 (41.5%)	25 (41.7%)	0.98
AV access number, n (%) 1 2 3 and more	57 (69.5%) 13 (15.8%) 12 (14.6%)	38 (63.3%) 11 (18.3%) 11 (18.3%)	0.73
Distal AVF, n (%)	69 (84.1%)	49 (81.7%)	0.30

*BMI-body mass index; RRT-renal replacement therapy; AVF-arteriovenous fistula; SBP-systolic blood pressure; DBP-diastolic blood pressure*.

On the study visit, a functioning AVF was found in 83 out of 157 (52.9%) patients. The AVF sites were as follows: snuffbox in six (7.2%), wrist in 41 (49.4%), distal forearm in 18 (21.7%), middle or proximal forearm in eight (9.6%), upper-arm AVG in one (1.2%), and upper-arm AVFs in nine (10.8%) patients, respectively. Blood flow ranged from 248 to 7,830 ml/min; the median was 1,134 ml/min. Median Qa was lower in patients with a diagnosis of diabetes mellitus compared to the patients without a comorbidity, 902 (492–1,362) vs. 1,185 (901–1,619) ml/min, *p* = 0.027. In 22 (26.5%) patients, Qa was higher than 1,500 ml/min. Patients with high-flow AVFs were younger than patients with AVFs with a Qa < 1,500 ml/min, 53.5 (48–59) vs. 61 (49–66) years old, *p* = 0.02. AVFs with a Qa > 1,500 ml/min were located more proximally (distal AVF in 68.2% in comparison to 88.5% in group with a lower Qa, *p* = 0.047). Mild symptoms of heart failure were observed in 25 (15.9%) KTx recipients. In symptomatic patients, the proportion of functioning AVF was similar compared to asymptomatic patients (60% vs. 51.5%, *p* = 0.43), Qa [1,135 (629–1,509) vs. 1,146 (827–1,551) ml/min, *p* = 0.71], and serum creatinine concentration [1.38 (1.15–1.75) vs. 1.35 (1.12–1.64) mg/dl, *p* = 0.75] were similar. However, the symptomatic patients were older [64 (61–69) vs. 55 (45–63) years old, *p* < 0.0001], the Charlson comorbidity index [5 (4–7) vs. 4 (3–5), *p* = 0.0003], and the prevalence of heart disease were higher (72 vs. 26.5%, *p* < 0.0001) compared to patients without symptoms of heart failure.

**Figure 1:**
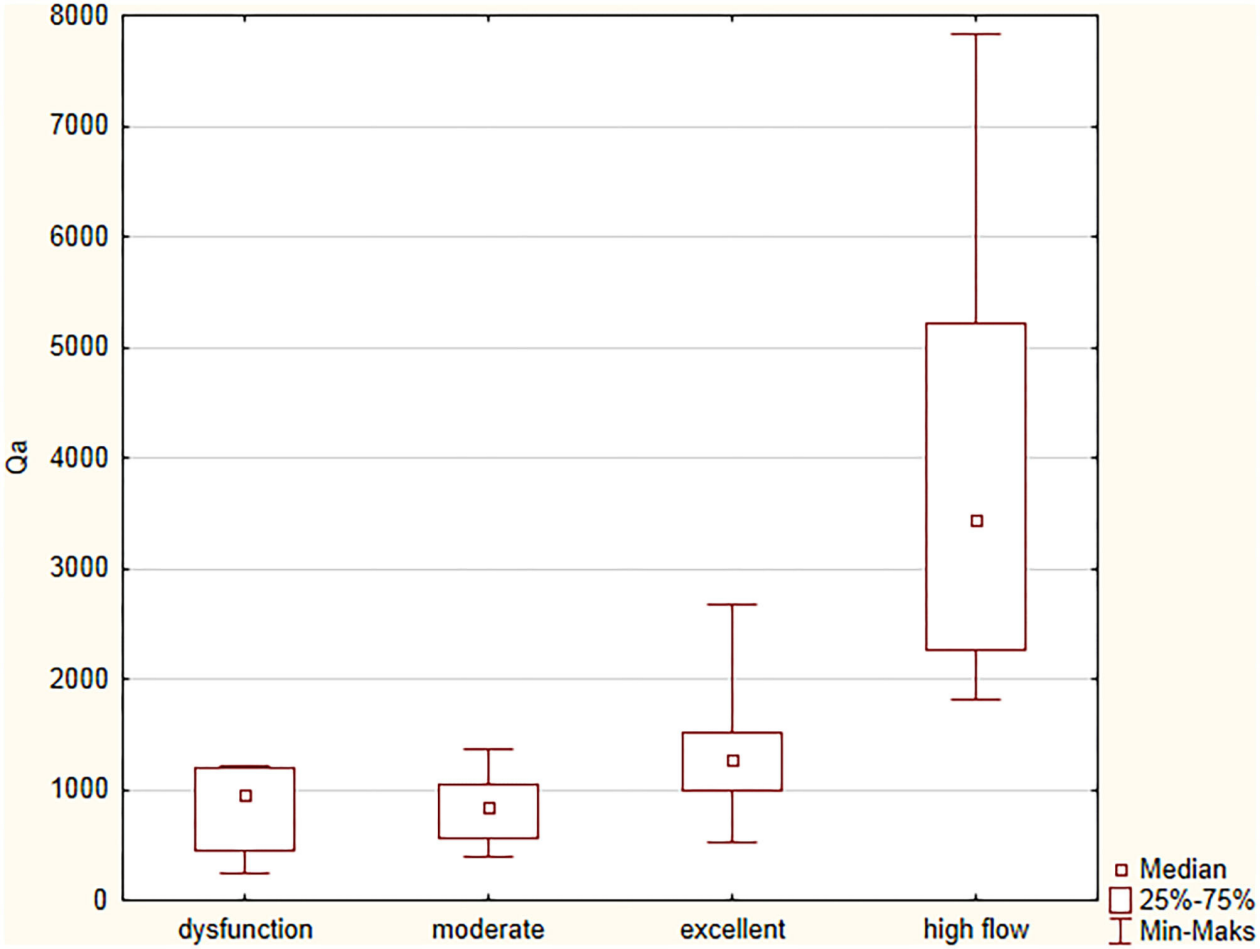
Vascular access flows in relation to AVF function assessed by physical examination.

On the basis of clinical assessment, VA were qualified to one of four groups: dysfunction, moderate, excellent function, and high-flow. The Qa values were different between groups; 957 (454–1,203), 847 (559–1,047), 1,279 (982–1,522), and 3,434 (2,258–5,223) ml/min, respectively, *p* < 0.0001 (Figure 1). In four patients, signs of cephalic arch stenosis were observed and two patients had complained from mild extremity edema.

We had noticed that 14 (8.9%) patients had problems with the vascular access creation or had very limited vascular access options. History of coronary angiography from the radial artery, subclavian vein catheterization, and presence of pacemaker were found in seven (4.4%), five (3.2%), and six (3.8%) patients, respectively. Proportion of patent VA in a group of patients more than 10 years after transplantation was lower than in patients with a shorter KTx vintage, 64 from 102 (62.7%) vs. 19 from 55 (34.5%) patients, *p* = 0.0007. The majority of patients (50 of 83, 60.2%) with an active AVF had options to create a snuffbox or wrist AVF on the contralateral extremity. In 76 out of 83 (91.6%) patients, the creation of a forearm AVF was possible. In a group of 74 patients without a functioning VA, the creation of a snuffbox or wrist AVF on the non-dominant and dominant extremity was possible in seven (9.2%) and 40 (52.6%) patients, respectively. In 10 (13.1%) patients, the possibilities were limited only to the upper-arm or proximal forearm on both sides. In a subgroup of 124 patients with one or two VA attempts, there were 71 (57.3%) functioning and 53 (42.7%) occluded AVFs in contrary to 29 patients with at least three VA attempts, there were 12 (41.4%) functioning and 17 (58.6%) occluded AVFs (*p* = 0.12). The proportions of distal AVFs were as follows: 89.6, 92.1, 41.7, and 35.3%, respectively (*p* < 0.0001). Distal AVF was an option in 66 (88%) and five (41.7%) patients (<3 vs. ≥3 VA attempts) contralateral to the functioning VA (*p* < 0.0001). In patients with an occluded AVF, a new VA could be created in the distal part of the dominant or non-dominant extremity in 46 (86.8%) and five (29.4%) patients (<3 vs. ≥3 VA attempts) (*p* < 0.0001), respectively. Access ligation was considered by 15 out of 83 (18.1%) patients with a patent AVF.

## Discussion

The approach to a functioning AVF in renal transplant recipients is ambiguous. Vascular access function is not routinely assessed in patients after renal transplantation. Routine VA surveillance was performed only by 29% of the responders to a survey investigating the preferences for management of VA after transplantation (9). A large proportion of patients with a failed graft reinitiating dialysis with catheter is a proof of suboptimal care in this area. Based on the data from the United States Renal Data System, it was found that in the first quarter after dialysis restart catheters were used in 53.1% of patients. AVF use was 37.4%, and it increased to 62.6% in the fourth quarter (10). In a Canadian cohort, starting dialysis after KTx failure, only 13%, 26%, and 38% of patients had an AV access creation in the postdialysis period at three, six, and 12 months, respectively (11). On the other hand, Manca et al. reported that 49 out of 89 patients restarted hemodialysis with the use of a still functioning AVF, and in the next 40 patients, a new VA had to be created (17). It is in line with the observation of Weyde et al. where a patent AVF that could be used immediately as VA was found in 49% of KTx recipients restarting dialysis. Proximal re-anastomosis on the forearm was successfully performed in 82 out of 112 patients (18). This approach resulted in a high prevalence of AVF in patients with a history of failed KTx (19).

In the present study, we sought to assess the quality of vascular access in a group of KTx recipients. In the majority of the studies in KTx patients, the function of vascular access was not considered except in focusing on its potential role. In those projects, usually only patients with high flow AVFs were included. We found that despite the distal location of AVF in the majority of our patients, the Qas were high but usually were below the threshold for hyperkinetic AVF. On the other side, the proportion of dysfunctional AVFs was low. Moreover, the majority of stenosis were located proximally, not close to the anastomosis as usual in the case of wrist AVFs. We were also able to identify patients who are at risk of a high-flow AVF on the basis of physical examination and also patients with limited vascular access perspectives. Finally, majority of the patients did not want to close the AVF. It is in line with our questionnaire-based study (8). It could be a consequence of our former view that the cardiovascular effect of a functioning AVF is mild and high prevalence of distal AVFs. We have to mention that the blood pressures recorded on the study visit were beyond the recommended values. The nature of this observation has to be explained but we suspect that it was related to the increased stress related to visits during the COVID-19 outbreak. Many patients participating in the study attended our transplantation office after missing a previously scheduled appointment. Moreover, visit to the clinic was related with filling questionnaires and crossing a few checkpoints. It is sure that the COVID-19 pandemic's impact on lifestyle factors, behaviors, and environmental changes are likely to have an influence on blood pressure control and cardiovascular risk (20).

Creation of AVF leads to hemodynamic effects like a decrease in blood pressure and total peripheral resistance, and an increase in heart rate, stroke volume, and cardiac output. After a few weeks, a blood volume and left ventricle diastolic diameter increase could occur. Further consequences of AVF creation are left ventricle hypertrophy, diastolic and systolic dysfunction, and increase in pulmonary flow and pulmonary hypertension (6, 21). Described effects are reversible after access closure, and are, at least partially, related to access flow volume (22–24). However, this correlation is not linear (25). It was shown that patients with a left ventricle hypertrophy (LVH) and left atrial enlargement had a worse outcome (26). Closure of AVF is an intervention that results in a decrease in LVH (22, 27). Routine closure of a functioning vascular access after successful KTx is not recommended in the guidelines and should be considered in patients with a refractory heart failure after transplantation (28). Recently, the effect of VA ligation on left ventricular mass was tested in the randomized study performed in stable KTx recipients. It was found that the removal of AVF improved the left ventricle remodeling and resulted in a reduction of NT-proBNP (27). However, it should be noted that in our study cohort, Qa and prevalence of upper arm AVFs were lower. In a recent study of Hetz et al. conducted in KTx patients with a Qa>1,500 ml/min., the prevention of right sided heart failure was noticed after VA ligature (29).

The further effect that could be influenced by fistula closure is the eGFR trajectory. In the observational study, it was found that patients with a persistent AVF at 1 year had a worse kidney function. They also had an increased risk for future allograft loss at 5 years (30). In contrary, Weekers et al. found that the closure of a patent VA significantly accelerated the eGFR decline over the 1-year observation (7). It was in line with the former observation of Golper who reported that the creation of AVF could slow the eGFR trajectory (31). The results of a recent meta-analysis demonstrated that AVF closure was related with an improved cardiac morphology, and a better kidney graft function and AVF closure had been suggested as a considerable approach in patients with a well-functioning allograft (32). Despite the modification of the cardiovascular risk through an intervention on AVF seems to be a promising intervention in KTx recipients, the patients were not convinced to a routine VA ligation (8). An alternative option could be a precision flow reduction by banding with a real-time access flow assessment or banding with the use of a sizing dowel (33–35). Using the data from the United States Renal Data Systems, it was found that AV access ligation was uncommon and performed in 4.6% of patients. Ligation was not associated with a post-transplant allograft failure and reduction in the all-cause mortality with AV access ligation. Based on literature data (36) and our own observation, we would not recommend a routine VA closure after KTx. More attention should be given to avoid the creation of high flow AVFs. The shift toward the creation of AVFs at the snuffbox seems to be the right direction (37, 38). We would also advise the routine evaluation of VA in KTx recipients at 1 year after transplantation to stratify the risk of a potential negative cardiovascular effect. Patients with previous multiple AVFs are at an increased risk of exhaustion of vascular access options. They should be very carefully assessed before every intervention on a functioning AVF and referred earlier for VA creation in case of a kidney graft failure progression. We are aware of the limitations of our study. First, it was a single center study. In addition, it involved a relatively small number of participants that constitutes about one eighth of our KTx patients and could not be a representative for the whole cohort. On the other hand, the baseline characteristic of our study group was similar in some aspects to previous reports (39–41). Finally, we believe that the problems of vascular access in KTx recipients were appropriately illustrated.

## Conclusions

Forearm AVF flows in kidney transplantation patients are below the threshold of the negative cardiovascular effect of vascular access. Creation of distal AVFs is a protective measure to avoid high flow and preserve vessels for future access. The approach to VA should be individualized and adjusted to the patient's profile. Doppler ultrasound assessment of a functioning AVF after a successful kidney transplantation is advisable.

## Data Availability Statement

The raw data supporting the conclusions of this article will be made available by the authors on reasonable request.

## Ethics Statement

The studies involving human participants were reviewed and approved by Institutional Ethical Committee at Wroclaw Medical University. The patients/participants provided their written informed consent to participate in this study.

## Author Contributions

KL contributed to the study concept, acquisition of data, analysis and interpretation of data, and preparation of the manuscript. AK, TG, HA-B, SZ, and MK-K contributed to the acquisition of data and revision of the manuscript. MB, OM, DK, and MK contributed to the study concept, design, and revision of the manuscript. All authors contributed to the article and approved the submitted version.

## Conflict of Interest

The authors declare that the research was conducted in the absence of any commercial or financial relationships that could be construed as a potential conflict of interest.

## References

[1] Woo K Lok CE. New insights into dialysis vascular access: what is the optimal vascular access type and timing of access creation in CKD and dialysis patients? Clin J Am Soc Nephrol. (2016) 11:1487–94. 10.2215/CJN.02190216 27401524 PMC4974877

[2] Letachowicz K Szyber P Gołebiowski T Kusztal M Letachowicz W Weyde W. Vascular access should be tailored to the patient. Semin Vasc Surg. (2016) 29:146–52. 10.1053/j.semvascsurg.2016.11.003 28779781

[3] Pisoni RL Zepel L Fluck R Lok CE Kawanishi H Süleymanlar G. International differences in the location and use of arteriovenous accesses created for hemodialysis: results from the dialysis outcomes and practice patterns study (DOPPS). Am J Kidney Dis. (2018) 71:469–78. 10.1053/j.ajkd.2017.09.012 29198387

[4] Robinson BM Akizawa T Jager KJ Kerr PG Saran R Pisoni RL. Factors affecting outcomes in patients reaching end-stage kidney disease worldwide: differences in access to renal replacement therapy, modality use, and haemodialysis practices. Lancet. (2016) 388:294–306. 10.1016/S0140-6736(16)30448-2 27226132 PMC6563337

[5] Airy M Lenihan CR Ding VY Montez-Rath ME Cheng J Navaneethan SD. Association between type of vascular access used in hemodialysis patients and subsequent kidney transplant outcomes. Kidney Med. (2019) 1:383–90. 10.1016/j.xkme.2019.08.005 32734218 PMC7384366

[6] Rao NN Dundon BK Worthley MI Faull RJ. The impact of arteriovenous fistulae for hemodialysis on the cardiovascular system. Semin Dial. (2016) 29:214–21. 10.1111/sdi.12459 26756565

[7] Weekers L Vanderweckene P Pottel H Castanares-Zapatero D Bonvoisin C Hamoir E. The closure of arteriovenous fistula in kidney transplant recipients is associated with an acceleration of kidney function decline. Nephrol Dial Transplant. (2017) 32:196–200. 10.1093/ndt/gfw351 27798197

[8] Bardowska K Letachowicz K Kamińska D Kusztal M Gołebiowski T Królicki T. The attitude of kidney transplant recipients towards elective arteriovenous fistula ligation. PLoS ONE. (2020) 15:e0234931. 10.1371/journal.pone.0234931 32615582 PMC7332306

[9] Voorzaat BM Janmaat CJ Wilschut ED Van Der Bogt KE Dekker FW Rotmans JI. No consensus on physicians' preferences on vascular access management after kidney transplantation: results of a multi-national survey. J Vasc Access. (2019) 20:52–9. 10.1177/1129729818776905 29843559 PMC6305957

[10] Zhang JC Al-Jaishi A Perl J Garg AX Moist LM. Hemodialysis arteriovenous vascular access creation after kidney transplant failure. Am J Kidney Dis. (2015) 66:646–54. 10.1053/j.ajkd.2015.03.031 25975965

[11] Huml AM Sehgal AR. Hemodialysis quality metrics in the first year following a failed kidney transplant. Am J Nephrol. (2019) 50:161–7. 10.1159/000501605 31311008 PMC6726525

[12] Abreo K Sachdeva B Abreo AP. To ligate or not to ligate hemodialysis arteriovenous fistulas in kidney transplant patients. J Vasc Access. (2020). 10.1177/1129729820970786. [Epub ahead of print]. 33176556

[13] Karim MS Aryal P Gardezi A Clark DF Aziz F Parajuli S. Vascular access in kidney transplant recipients. Transplant Rev. (2020) 34:100544. 10.1016/j.trre.2020.100544 32205010

[14] Lok CE Huber TS Lee T Shenoy S Yevzlin AS Abreo K. KDOQI clinical practice guideline for vascular access: 2019 update. Am J Kidney Dis. (2020) 75(Suppl. 2):S1–164. 10.1053/j.ajkd.2019.12.001 32778223

[15] Salman L Beathard G. Interventional nephrology: physical examination as a tool for surveillance for the hemodialysis arteriovenous access. Clin J Am Soc Nephrol. (2013) 8:1220–7. 10.2215/CJN.00740113 23824199

[16] Mudoni A Caccetta F Caroppo M Musio F Accogli A Zacheo MD. Echo color Doppler ultrasound: a valuable diagnostic tool in the assessment of arteriovenous fistula in hemodialysis patients. J Vasc Access. (2016) 17:446–52. 10.5301/jva.5000588 27470250

[17] Manca O Pisano GL Carta P Manca EM Piredda GB Pili G. The management of hemodialysis arteriovenous fistulas in well**-**functioning renal transplanted patients: many doubts, few certainties. J Vasc Access. (2005) 6:182–6. 10.1177/112972980500600405 16552699

[18] Weyde W Letachowicz W Krajewska M Gołebiowski T Letachowicz K Kusztal M. Arteriovenous fistula reconstruction in patients with kidney allograft failure. Clin Transplant. (2008) 22:185–90. 10.1111/j.1399-0012.2007.00767.x 18339138

[19] Letachowicz K Weyde W Letachowicz W Klinger M. The effect of type and vascular access quality on the outcome of chronic hemodialysis treatment. J Ren Nutr. (2010) 20:S118–21. 10.1053/j.jrn.2010.06.012 20797559

[20] Kreutz R Dobrowolski P Prejbisz A Algharably EAE Bilo G Creutzig F. Lifestyle, psychological, socioeconomic and environmental factors and their impact on hypertension during the coronavirus disease 2019 pandemic. J Hypertens. (2020). 10.1097/HJH.0000000000002770. [Epub ahead of print]. 33395152

[21] Duque JC Gomez C Tabbara M Alfonso CE Li X Vazquez-Padron RI. The impact of arteriovenous fistulae on the myocardium: the impact of creation and ligation in the transplant era. Semin Dial. (2015) 28:305–10. 10.1111/sdi.12313 25267110

[22] Dundon BK Torpey DK Nelson AJ Wong DT Duncan RF Meredith IT. Beneficial cardiovascular remodeling following arterio-venous fistula ligation post-renal transplantation: a longitudinal magnetic resonance imaging study. Clin Transplant. (2014) 28:916–25. 10.1111/ctr.12402 24931318

[23] Vanderweckene P Weekers L Lancellotti P Jouret F. Controversies in the management of the haemodialysis-related arteriovenous fistula following kidney transplantation. Clin Kidney J. (2018) 11:406–12. 10.1093/ckj/sfx113 29992020 PMC6007507

[24] Cridlig J Selton-Suty C Alla F Chodek A Pruna A Kessler M. Cardiac impact of the arteriovenous fistula after kidney transplantation: a case-controlled, match-paired study. Transpl Int. (2008) 21:948–54. 10.1111/j.1432-2277.2008.00707.x 18537919

[25] Basile C Lomonte C Vernaglione L Casucci F Antonelli M Losurdo N. The relationship between the flow of arteriovenous fistula and cardiac output in haemodialysis patients. Nephrol Dial Transplant. (2008) 23:282–7. 10.1093/ndt/gfm549 17942475

[26] Patel RK Pennington C Stevens KK Taylor A Gillis K Rutherford E. Effect of left atrial and ventricular abnormalities on renal transplant recipient outcome-a single-center study. Transplant Res. (2014) 3:20. 10.1186/s13737-014-0020-6 25505546 PMC4261520

[27] Rao NN Stokes MB Rajwani A Ullah S Williams K King D. Effects of arteriovenous fistula ligation on cardiac structure and function in kidney transplant recipients. Circulation. (2019) 139:2809–18. 10.1161/CIRCULATIONAHA.118.038505 31045455

[28] Schmidli J Widmer MK Basile C de Donato G Gallieni M Gibbons CP. Editor's choice - vascular access: 2018 clinical practice guidelines of the European society for vascular surgery (ESVS). Eur J Vasc Endovasc Surg. (2018) 55:757–818. 10.1016/j.ejvs.2018.02.001 29730128

[29] Hetz P Pirklbauer M Müller S Posch L Gummerer M Tiefenthaler M. Prophylactic ligature of AV fistula prevents high output heart failure after kidney transplantation. Am J Nephrol. (2020) 51:511–9. 10.1159/000508957 32659755 PMC7592949

[30] Vajdič B Arnol M Ponikvar R Kandus A Buturović-Ponikvar J. Functional status of hemodialysis arteriovenous fistula in kidney transplant recipients as a predictor of allograft function and survival. Transplant Proc. (2010) 42:4006–9. 10.1016/j.transproceed.2010.09.057 21168612

[31] Golper TA Hartle PM Bian A. Arteriovenous fistula creation may slow estimated glomerular filtration rate trajectory. Nephrol Dial Transplant. (2015) 30:2014–8. 10.1093/ndt/gfv082 25888388 PMC4832989

[32] Zheng H Bu S Song Y Wang M Wu J Chen J. To ligate or not to ligate: a meta-analysis of cardiac effects and allograft function following arteriovenous fistula closure in renal transplant recipients. Ann Vasc Surg. (2020) 63:287–92. 10.1016/j.avsg.2019.06.040 31536798

[33] Letachowicz K Kusztal M Gołebiowski T Letachowicz W Weyde W Klinger M. External dilator-assisted banding for high-flow hemodialysis arteriovenous fistula. Ren Fail. (2016) 38:1067–70. 10.1080/0886022X.2016.1184936 27185420

[34] Gkotsis G Jennings WC Malik J Mallios A Taubman K. Treatment of high flow arteriovenous fistulas after successful renal transplant using a simple precision banding technique. Ann Vasc Surg. (2016) 31:85–90. 10.1016/j.avsg.2015.08.012 26616507

[35] Jennings WC Nelson PR. The role of precision banding of arteriovenous fistulas in successful kidney transplant recipients. J Vasc Surg. (2020) 71:719. 10.1016/j.jvs.2019.10.026 32040439

[36] Hicks CW Bae S Pozo ME DiBrito SR Abularrage CJ Segev DL. Practice patterns in arteriovenous fistula ligation among kidney transplant recipients in the United States renal data systems. J Vasc Surg. (2019) 70:842–52.e1. 10.1016/j.jvs.2018.11.048 30853386

[37] Letachowicz K Gołebiowski T Kusztal M Letachowicz W Weyde W Klinger M. The snuffbox fistula should be preferred over the wrist arteriovenous fistula. J Vasc Surg. (2016) 63:436–40. 10.1016/j.jvs.2015.08.104 26602796

[38] Idrees M Suthananthan A Pathmarajah T Sieunarine K. Snuffbox fistula – a first-line approach to haemodialysis: a review. J Vasc Access. (2020) 21:554–63. 10.1177/1129729819867817 31419923

[39] Letachowicz K Królicki T Bardowska K Drabik A Zajdel K Kamińska D. The impact of functioning arteriovenous fistula on blood pressure control and renal allograft function. Transplant Proc. (2018) 50:1855–7. 10.1016/j.transproceed.2018.03.109 30056915

[40] Heleniak Z Illersperger S Brakemeier S Debska-Slizień A Budde K Halleck F. Obesity, fat tissue parameters, and arterial stiffness in renal transplant recipients. Transplant Proc. (2020) 52:2341–6. 10.1016/j.transproceed.2020.01.118 32444129

[41] Letachowicz K Bardowska K Królicki T Kamińska D Banasik M Zajdel K. The impact of location and patency of the arteriovenous fistula on quality of life of kidney transplant recipients. Ren Fail. (2021) 43:113–22. 10.1080/0886022X.2020.1865171 33397180 PMC7801108

